# Si solid-state quantum dot-based materials for tandem solar cells

**DOI:** 10.1186/1556-276X-7-193

**Published:** 2012-03-21

**Authors:** Gavin Conibeer, Ivan Perez-Wurfl, Xiaojing Hao, Dawei Di, Dong Lin

**Affiliations:** 1 ARC Photovoltaics Centre of Excellence, University of New South Wales, Sydney, NSW 2052, Australia

**Keywords:** band gap engineering, quantum dots, photovoltaics, tandem cells, modulation doping, nucleation, third generation

## Abstract

The concept of third-generation photovoltaics is to significantly increase device efficiencies whilst still using thin-film processes and abundant non-toxic materials. A strong potential approach is to fabricate tandem cells using thin-film deposition that can optimise collection of energy in a series of cells with decreasing band gap stacked on top of each other. Quantum dot materials, in which Si quantum dots (QDs) are embedded in a dielectric matrix, offer the potential to tune the effective band gap, through quantum confinement, and allow fabrication of optimised tandem solar cell devices in one growth run in a thin-film process. Such cells can be fabricated by sputtering of thin layers of silicon rich oxide sandwiched between a stoichiometric oxide that on annealing crystallise to form Si QDs of uniform and controllable size. For approximately 2-nm diameter QDs, these result in an effective band gap of 1.8 eV. Introduction of phosphorous or boron during the growth of the multilayers results in doping and a rectifying junction, which demonstrates photovoltaic behaviour with an open circuit voltage (*V*_OC_) of almost 500 mV. However, the doping behaviour of P and B in these QD materials is not well understood. A modified modulation doping model for the doping mechanisms in these materials is discussed which relies on doping of a sub-oxide region around the Si QDs.

## Introduction

An increase in efficiency can be achieved through the use of multiple energy levels in a third-generation photovoltaic device. One configuration which increases the voltage output from the device is the tandem or multi-junction cell, in which cells with increasing band gap are stacked on top of each other such that each cell absorbs a different part of the solar spectrum with a narrower range and hence more optimally for each absorbed photon. The sum of the output from these cells can boost the overall efficiency. In practice, it is easiest to configure the cells such as to have the same current through each cell in an in-series or current-matched two-terminal device.

**Figure 1:**
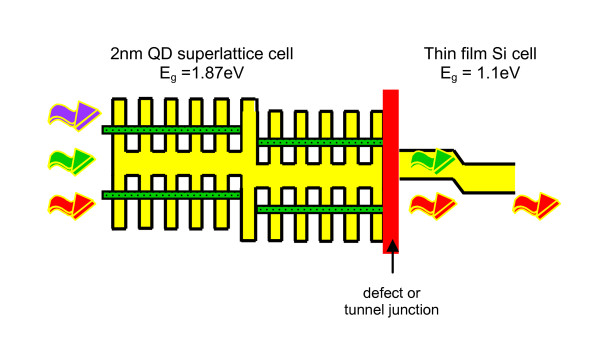
**Si nanostructure/Silicon tandem cell**. The nanostructure cell consists of a multilayer structure of Si QDs in an amorphous dielectric matrix, with a junction between p-type and n-type material, all connected by a defect tunnel junction to a thin-film Si cell.

The Si quantum dot (QD) solar cell is suggested as a way to engineer the band gap (*E*_g_) of the top cell or cells in a tandem stack using thin-film deposition methods [1-3]. The very small scale of QDs of a few nanometers, and hence less than the Bohr radius of Si, results in increased confined energy levels through quantum confinement. For QDs the effect is stronger than quantum wells because confinement is in all three dimensions. For QDs which are close to each other such that there is a significant tunnelling probability, a true miniband will form and result in an increase in the effective band gap of the tailored superlattice material. The conceptual design of a complete device is shown in Figure 1.

Incorporation of P or B during growth results in n- or p-type materials, respectively, and allows growth of rectifying junctions, which have photovoltaic properties. Si QD devices have been demonstrated by some of the current authors with *V*_OC _up to 490 mV [4-7]. However, further optimisation of these materials and devices such as to increase *V*_OC _and improve the presently very low current densities (tens of microamperes per square centimetre) requires a better understanding of the exact mechanisms of doping.

## Fabrication of Si QD nanostructures

Structures are grown using magnetron sputtering on quartz substrates. Alternating layers of a Si-rich dielectric and a stoichiometric layer of dielectric are deposited. Layer thicknesses are kept constant within one structure, although the stoichiometric dielectric may well have a different thickness to the Si-rich layer. Layer thicknesses are varied from sample to sample between 2 and 7 nm. The number of bi-layers is also varied but is typically between 20 and 40. The dielectric used is usually SiO_2_, although Si_3_N_4 _and SiC are also used. This layer growth technique for SiO_2 _was borrowed from [8] and adapted for photovoltaics by [1].

**Figure 2:**
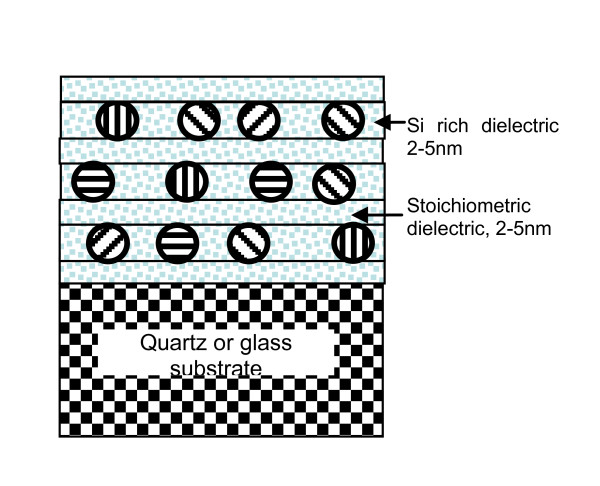
**Multilayer deposition of alternating Si-rich dielectric and stoichiometric dielectric in layers of a few nanometers**. On annealing, the excess Si precipitates out to form small nanocrystals of a size determined by the Si-rich layer thickness. The nanocrystal or quantum dot size is therefore uniform.

Post-deposition, the structures are annealed typically at 1,050 to 1,150°C for 1 to 2 h. During this process, the high temperature nucleates the growth of small nanocrystals from the excess Si in the Si-rich layers. These nanocrystals grow depending on the growth time until all the excess Si has diffused to growing nanocrystals. The size of these nanocrystals is limited by the border with the stoichiometric dielectric region in which there is no excess Si to diffuse. This results in a fairly uniform nanocrystal size which is desirable for the formation of quantum dots with fairly uniform confined energy level as it increases the probability of formation of a true miniband. This process is shown schematically in Figure 2.

For Si QDs in SiO_2_, the precipitation occurs according to the following:

(1) $$\text{Si}{\text{O}}_{x}\to \frac{x}{2}\text{Si}{\text{O}}_{2}+(1-\frac{x}{2})\text{Si}$$

Precipitation of excess Si from Si-rich dielectrics in SiN*_x _*and SiC*_x _*follows a similar crystallisation reaction as Si precipitates from the amorphous matrix.

The decreasing barrier height on going from SiO_2 _to Si_3_N_4 _to SiC is used to increase the tunnelling probability between adjacent quantum dots or between quantum dot layers. Si_3_N_4 _and SiC give lower barriers than SiO_2 _allowing larger dot spacing for a given tunnelling current. The wave function of an electron confined to a spherical dot penetrates into the surrounding material, decreasing exponentially into the barrier. The slope of this exponential decay and hence the barrier to tunnelling between quantum dots is reduced for a lower barrier height material. This is because - from transmission/reflection probability - the tunnelling probability *T*_e _through a square potential well depends exponentially on three parameters, the barrier width, *d *(equal to the spacing between quantum dots); the square root of the barrier height seen by the electron (Δ*E*^1/2^, the energy difference between the CB edge of the matrix and the confined energy level of the quantum dots, equivalent to (*E*_c_-*E*_n_)^1/2^); and the square root of the effective mass (m*)^1/2 ^of the electron in the barrier. This gives the approximate relation (e.g. [9], p. 244):

(2) $$T_{\text{e}}\approx 16\text{exp}\left\{-d\sqrt{\frac{8m^{*}}{\hbar^{2}}\;\text{Δ}E}\right\}$$

Fluctuations in spacing and size of the dots around their mean values can be investigated using similar calculations. Using this approach, it is also found that the calculated Bloch mobilities do not depend strongly on variability in the dot position around a mean position, *Δd*, but do depend strongly on variation in the dot size within the QD material [10]. This is an important result for engineering a real thin-film structure, because although it is necessary to have a reasonably uniform QD size and to minimise the *mean *spacing between QDs, *d*, to give high mobilities for a given matrix, the variation around this mean value, *Δd*, is less critical. Hence, transport between dots can be significantly increased by using alternative matrices with a lower barrier height, *ΔE*.

## Optical absorption properties of Si nanostructures

**Figure 3:**
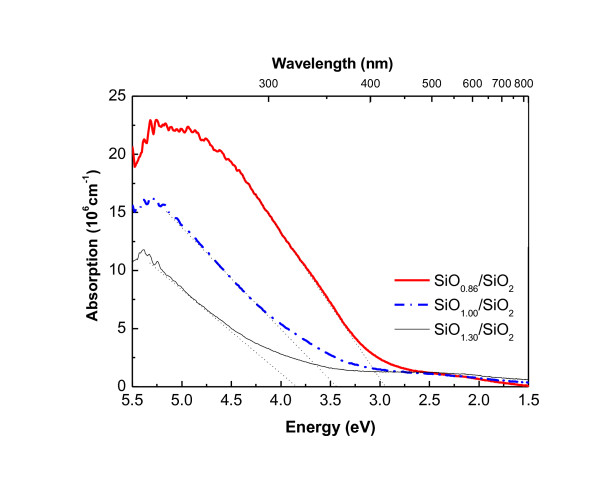
**Room temperature absorption coefficient of annealed SiO*_x_*/SiO_2 _multilayer films with various *x *(1,100°C, 1 h; **[11]). There is a strongly absorbing region at energies above 3 eV, in which absorption increases with the Si content of the layers, and a weakly absorbing region at less than 3 eV, which is not strongly affected by Si content.

The measured optical absorption for Si QD in SiO_2 _materials, for multilayered Si nanostructured material of various Si content, is shown in Figure 3[11]. There is a clear region of high absorption coefficient for photon energies above about 3 eV. As the Si content of the layers increases (i.e. as *x *decreases) the strength of this absorption increases. This is because there is more Si to absorb photons, most of which is bound up in precipitated Si QDs. In addition to this onset of strong absorption, it is clear from Figure 3 that there is also a significant weak absorption tail below 3 eV. This does not have a dependence on Si content.

This suggests the presence of a weakly absorbing background surrounding the crystalline Si QDs. The small dependence of absorption of this region on the Si content suggests it is a sub-stoichiometric oxide with only a very small amount of O. The identification of this sub-oxide region is important for the possible modulation doping of this region by incorporated P or B atoms, as discussed below in 'Doping mechanisms for Si QD superlattice structures' section, as a theory to explain doping behaviour.

Further evidence for the presence of such a sub-oxide region is given by a study by Daldosso et al. for very similar Si nanocrystals material in SiO_2 _[12]. In this work, experimental data on X-ray absorption and energy-filtered TEM clearly show the presence of amorphous Si sub-stoichiometric oxide regions around Si nanocrsystals, with these regions extending for at least 1 nm. They further use *ab initio *methods to model the energy levels associated with an interface between nanocrystals and a stoichiometric SiO_2 _matrix which has an influence on absorption and light emission properties. This is very consistent with the sub-oxide region proposed around Si nanocrystals in the current work.

## Doping and device fabrication for QD nanostructures

Doping in these multilayer QD structures is achieved by incorporation of either phosphorous pentoxide for n-type doping or boron for p-type doping into the Si-rich material by co-sputtering during growth of the appropriate layers. To obtain a p-n junction, several layers (typically 20) doped with P_2_O_5 _are followed by several more (again typically 20) doped with B, sometimes with a few undoped layers in-between. The sample is then annealed at approximately 1,100°C to form Si QDs and to activate these dopants. Typically, hydrogenation is then performed in a cold-wall vacuum system at 600-625°C for 15 min.

p-n junction devices, fabricated in this way, exhibit photovoltaic properties [5]. Current-voltage (*I*-*V*) measurements in the dark and under 1-sun illumination indicate a good rectifying junction and generation of an open circuit voltage, *V*_OC_, up to 492 mV [4]. The high sheet resistance of the deposited layers, in conjunction with the insulating quartz substrates, causes an unavoidable very high series resistance in the devices. The high resistance severely limits both the short circuit current and the fill factor of the cells, particularly under illumination. It also makes it necessary to include effects due to in-plane current flow in the analysis of the measured electrical characteristics [6].

**Figure 4:**
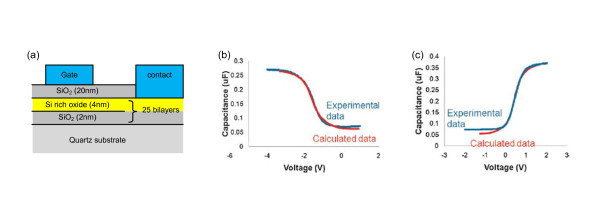
**MOS samples, B-doped Si-rich oxide and P-doped Si-rich oxide**. MOS samples as shown in (**a**), with *C*-*V *curve fitting for (**b**) B-doped Si-rich oxide showing accumulation for negative bias and hence p-type behaviour and (**c**) P-doped Si-rich oxide showing inversion for negative bias and hence n-type behaviour. The calculated curves use an MOS model with p- and n-type material respectively. The clear change of sign shows the opposite carrier type for B and P doping [14].

In related works, very significant decreases in resistivity have been observed in B- [13] and P-doped [5] materials, with related reductions in activation energy from resistance with temperature measurements. Further strong evidence for doping is shown in [14]*C*-*V *data for metal-oxide semiconductor (MOS) structures grown using Si-rich oxide multilayer structures as shown schematically in Figure 4a. The *C*-*V *data for Boron-doped silicon-rich oxide (SRO) in Figure 4b show that for a negatively biased gate, the sample exhibits accumulation, indicating p-type behaviour as a significant capacitance accumulates across the 20 nm oxide layer. Whereas for phosphorous-doped SRO Figure 4c shows inversion for the same negative bias on the gate, indicating n-type behaviour with minimal accumulation across the oxide. The modelled fit to these data is for a 4 × 10^17^-cm^-3 ^hole and 1 × 10^17^-cm^-3 ^electron concentration for B and P doping, respectively. These data clearly show that p-type and n-type doping occur on incorporation of B and P respectively, although the exact nature of doping in these QD structures is still the subject of intense theoretical and experimental work [15].

## Doping mechanisms for Si QD superlattice structures

As discussed in "Doping and device fabrication for QD nanostructures" section, there is significant evidence for doping on incorporation of B and P in Si QD nanostructures from resistivity data and from the formation of rectifying p-n junctions which behave as photovoltaic devices. However, the mechanisms for this doping are not clear. Direct doping of the QDs by impurity atoms is extremely unlikely as the perfectly crystalline nanocrystals segregate any impurity atoms during growth, as shown in *ab initio *calculations [16,17]. Experimentally, this is supported by data on the free electron density in Si nanocrystals using electron paramagnetic resonance (EPR) [18] which show that at least 95% of P atoms are inactive and segregated to the surface of Si nanocrystals, such that their contribution to doping is at least an order of magnitude lower than their atomic concentration.

Another possibility for doping is that the matrix material, rather than the QDs, is doped by the impurity atoms and that this then provides free carriers which are captured by the QDs. This 'modulation doping' mechanism is commonly used in III-V nanostructures. But in III-Vs, the barrier height is typically only a few 100 meV and hence ionisation of dopant atoms is relatively easy [9]. In the dielectric matrices used in the Si QD work (particularly for the SiO_2 _barriers), the barrier height is several electronvolts. Under these circumstances, there would be no ionisation of dopant atoms at room temperature and hence modulation doping could only occur by direct tunnelling of carriers from dopants in SiO_2 _less than 1 nm from the QDs.

**Figure 5:**
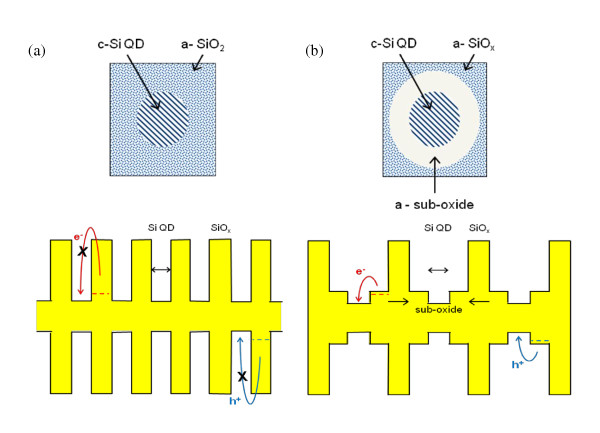
**Schematic illustration of "modified modulation" doping**. (**a**) QDs dispersed in a stoichiometric SiO_2 _matrix - dopant atoms cannot be ionised as they create very deep levels. (**b**) QDs surrounded by an amorphous sub-oxide region between regions of full stoichiometric barrier material - dopant atom levels are shallow enough to be ionised at room temperature and 'modulation' dope the QDs.

If direct doping or doping of a stoichiometric SiO_2 _matrix are not feasible, then a third possibility is that it is the sub-oxide region surrounding the QDs, as discussed in "Optical absorption properties of Si nanostructures" section and suggested in [12], which is being modulation doped by the P or B dopant atoms. If this region is a sufficiently extended silicon sub-oxide (SiO*_x_*), it could provide flat Bloch bands able to be doped in a pseudo bulk-like regime. If, in addition, *x *in the sub-oxide is less than about 0.5 (as the data of Figure 3 suggests), then the consequent relatively small band gap would allow B and P doping levels shallow enough to be ionised at room temperature. The resultant free electrons in the case of P doping (or holes for B doping) would then be available to be captured by the QDs. This would raise (lower) the Fermi level in the QD superlattice and hence create n-type (p-type) material. Alternatively, or perhaps as well, electrons (holes) freed by ionisation could remain in the sub-oxide raising (lowering) its Fermi level and hence directly doping the sub-oxide itself. This modified modulation doping mechanism is illustrated schematically in Figure 5.

This explanation remains a hypothesis at present. More evidence on the exact positioning of dopant atoms is required from techniques such as atom probe tomography together with direct evidence of doping from EPR.

## Conclusions

Quantum confinement in Si QD nanostructures is proposed to engineer an increase in the effective band gap and hence produce materials suitable for upper cell elements in a thin-film tandem cell. Si QD materials grown by sputtering alternating Si-rich oxide and stoichiometric SiO_2 _layers, with a subsequent anneal to precipitate a layered growth of QDs, have been fabricated in previous work. Absorption data on these show a strong absorption region with absorption dependent on Si content and a significant weak absorption tail attributed to sub-oxide regions surrounding QDs due to incomplete diffusion to the growing QDs. Dramatic decreases in resistivity have been demonstrated on incorporation of P or B dopants, and rectifying p-n junctions have been fabricated which give a *V*_OC _up to 490 mV. However, the mechanism of doping in these materials can neither be direct doping of the QDs nor modulation doping of a stoichiometric SiO_2 _matrix. A modified modulation doping mechanism is proposed in which P or B atoms dope the sub-oxide region shown to surround QDs, which because of its reduced band gap has dopant levels shallow enough to be ionised and hence provide free carriers to the QDs.

## Competing interests

The authors declare that they have no competing interests.

## Authors' contributions

GC worked on the initial concept, on the theory of modified modulation doping and drafted the manuscript. XH carried out the preparation, doping and characterisation of engineered band gap materials; IP fabricated devices and characterised doping and photovoltaic properties; DD participated in the device fabrication and some of the materials preparation; DL fabricated and characterised doped MOS samples. All authors read and approved the final manuscript.
